# Pathogenicity study of Iranian genotype of avian infectious bronchitis virus (IR-1)

**Published:** 2017-03-15

**Authors:** Hamideh Najafi, Arash Ghalyanchi Langeroudi, Masoud Hashemzadeh, Vahid Karimi, Omid Madadgar, Reza Khaltabadi Farahani, Seyed Ali Ghafouri, Hossein Maghsoudloo, Parvaneh Seifouri, Ali Madhi

**Affiliations:** 1 *Department of Microbiology and Immunology; Faculty of Veterinary Medicine, University of Tehran, Tehran, Iran;*; 2 *Department of Research and Production of Poultry Viral Vaccine, Razi Vaccine and Serum Research Institute, Karaj,Iran;*; 3 *Department of Poultry Diseases, Faculty of Veterinary Medicine, University of Tehran, Tehran, Iran;*; 4 *Iranian veterinary Organization, Tehran, Iran.*

**Keywords:** Avian infectious bronchitis, Biochemical analysis, Histopathology, Pathogenesis, Real-time PCR

## Abstract

Avian infectious bronchitis (IB) is a major cause of economic losses in poultry industry. The IB virus primarily affects respiratory tract, but various strains differ in their tropism for other target organs such as kidney and alimentary tract. The objective of this study was to estimate the pathogenicity of Iranian IBV variant (IR-1), which is limited exclusively to Iran. Specific pathogen free chicks were inoculated intranasally. Sera, fecal swabs and different tissue samples were collected on different days post infection (DPI). Clinical signs, gross pathology and histological changes were recorded. The viral load was quantified in the RNA extractions from different tissue samples using real-time PCR. Anti-IBV antibodies were detected in serum samples. The IgG antibody were found on 21 and 28 DPI. Severe histological lesions were observed in the trachea and lung while the lesions in kidney were appeared to be milder. Viral RNA was detected in all tested tissues from 1 DPI to the last day of the experiment. The highest viral load was measured in the trachea and feces on 1^st^ and 5^th^ DPI, respectively. It can be concluded the IR-1 had broad tropism for respiratory tract, digestive system, and renal tissue, reflecting its epitheliotropic nature, but it caused the most severe lesions in the respiratory tract. This was the first pathogenicity study of Iranian IR-1 IBV. Further knowledge of IBV pathogenesis provides the groundwork to inform more effective prevention practices.

## Introduction

Infectious bronchitis (IB) is a globally distributed avian disease that represents one of the most persistent sanitary problems in the commercial poultry industry. The etiologic agent, infectious bronchitis virus (IBV), belongs to the genus *Gammacoronavirus *within the *Coronaviridae *family.^1^^,^^2^ The disease is characterized by respiratory-specific symptoms including gasping, coughing, sneezing, tracheal rales and nasal discharge.^3^ Chicks of all ages are susceptible to infection and the virus replicates in many tissues including the trachea, lung, spleen, kidney and ovaries. It is recognized that IBV isolates differ in virulence and tissue tropism.^4^ Most IBV strains are respiratory, inducing histological lesions in the trachea.^5^ However, Winterfield and Albassam noted that various passage levels of an IB vaccine virus, the Holland strain, were capable of causing microscopic lesions of interstitial nephritis.^6^ In addition, nephropathogenic strains cause histological lesions in trachea and kidneys.^7^^-^^9^ The first isolation of IBV from Iranian chick flocks was reported in 1994. Later, several Iranian researchers identified the 793/B serotype. This serotype turned out to be one of the predominant types of IBV in Iran.^10^ Different vaccines (Mass and 793/B vaccines) are now widely used in Iranian poultry farms, but IB outbreaks are still occurring. Recently, one of IBV variants named as IR-1, which was characterized based on partial S1 gene sequencing, has been isolated from Iranian broiler farms. Hashemzadeh *et al*. investigated the phylogenetic relation-ship of Iranian IBV isolates during 2010 to 2011. Moreover, they identified a new IBV variant, which was not related to any identified genotypes of IBV.^11^ This new IBV was again detected in a molecular surveillance performed during 2010 to 2014 and designated IR-1.^12^ Thereafter, IR-1 was isolated from the tissue samples collected from Iranian broiler farms during 2014 to 2015.^13^ The IR-1 which has been detected in 2010, is only circulated in Iranian poultry farms. In addition, there is no information about IR-1 pathogenic aspects. The aim of the present study was to evaluate IR-1 pathogenicity and tissue tropism in experimentally infected specific pathogen free (SPF) chicks. 

## Materials and Methods

**Strain history and virus titration. **The IR-1 genotype of IBV (accession number: KT583580), isolated from Iranian broiler farms in 2014 was used for examining its pathogenicity. The Reed-Muench method^14^ was used to calculate 50% egg infective dose (EID_50_) and the isolate was used in the experimental pathogenicity study.

**Experimental design. **A total number of 49 SPF 1-day-old chicks were divided randomly into two groups, 35 chicks in the experimental and 14 chicks in the control group, Each group was housed in a separate isolator. At the age of 14 days, birds in the experimental group were challenged intranasally with the allantoic fluid of IR-1 containing 10^4^ EID_50_ per 0.10 mL of the virus. Up to 28 days post infection (DPI) chicks were observed and clinical signs and mortality were recorded. Clinical signs were scored as follow: 0 = no abnormal breathing; 1 = slight or rare tracheal rales can be heard after provoking the chick by moving; 2 = moderate or frequent tracheal rales can be heard without moving the chick but not continuously and 3 = marked, continuous tracheal rales can be heard. On 1, 3, 5, 7, 14, 21, and 28 DPI, five chicks from the experimental group and two chicks from control group were randomly selected, the serum was collected for an IB ELISA test. On days 1, 3, 5, 7, 14 and 21, tissue samples of the trachea, lung, spleen, kidneys, proventriculus, cecal tonsils and cloacal swabs were aseptically collected for virus quantification using real-time PCR.

**Detection of anti-IBV antibodies. **The IBV antibody test kit, ProFLOK IBV ELISA kit (Synbiotics Corporation, San Diego, USA), was used for the detection of a specific antibody to IBV in the serum samples according to manufacturer’s instruction. 

**Biochemical analysis. **The level of uric acid, creatinine, alanine amino transferase activity and aspartate amino transferase activity in the serum samples were measured using standard auto analyzer Selectra ProXSwith software (version 1.0.X; Vital Scientific BV, Gelderland, The Netherlands). 

**Primers and probes. **Real-time PCR was used to amplify a conserved sequence within the 5′-untranslated region (UTR) of IBV genome and 28s rRNA was used as a reference gene. To amplify a 143-bp fragment of the 5′-UTR of the IBV genome, we used a downstream primer (IBV5′ GU391 5′-GCTTTTGAGCCTAGCGTT-3′, located at nucleotide position 391-408 of the IBV M41 genome sequence), an upstream primer (IBV5′GL533 5′-GCCATGTTGTCACTGTC TATTG-3′, located at nucleotide position 512-533 of the IBV M41 genome sequence) and Taqman^®^ dual-labeled probe (IBV5′G probe 5′-FAMCACCACCAGAACCTGTCACCT C-BHQ1-3′, located at nucleotide position 473-494) which were designated previously.^15^ For the 28s rRNA gene, the forward primer (28s F 5′GGCGAAGCCAGA GGAAACT-3′), reverse primer (28s R 5′GACGACCGATTTGC ACGTC-3′) and probe (FAM-AGGACCGCTACGGACCTCCACCA-TAMRA) were used to amplify a 61 bp fragments of the gene.^16^

**The IBV quantitation standards. **The 5' UTR of M41 reference IBV strain and 28s rRNA of chick tissue were amplified using the same primers applied in real-time PCR reaction. The PCR products were examined using agarose-gel electrophoresis. After ethidium bromide staining, the bands were visible only at the expected molecular weights (a 143-bp fragment for 5′-UTR of M41 and 61 bp fragment for 28srRNA). The PCR products for 5′-UTR and 28s rRNA were cloned in a pTG19-T vector and transformed in *Escherichia Coli* TOP10 competent cells. Plasmid isolation kit mini-preparation (Molecular biological system transfer, Tehran, Iran) was used to extract plasmids. Before use, the plasmid concentration was determined by spectro-photometry at 260 nm and calculated as genome equivalents (copies) per mL as the molecular weight of the plasmid was known. Serial dilutions were performed to give a final concentration between 10^2^ and 10^5^ (for 5′-UTR) or 10^2^ and 10^5^ (for 28s rRNA) copies for generating the standard curves. 

**The RNA extraction and cDNA synthesis. **The RNA was extracted from tissue samples using the Cinna Pure RNA (SinaClon, Tehran, Iran) kit following instructions and treated with Dnaes I. The cDNA was generated using RevertAid First Strand cDNA Synthesis kit (Thermo Fisher Scientific Inc., Waltham, USA).

**Real-time PCR. **The 20 μL real-time PCR reaction contained 2 μL 10X PCR buffer (SinaClon), 1 μL dNTP mix (10 mM; SinaClon), 0.80 μL Mgcl_2_ (50 mM; SinaClon), 0.20 μL CinnaGen *Taq* DNA polymerase (1 U) (SinaClon), 5 μL template cDNA, primers at a final concentration of 0.10 μM, probe at a final concentration of 0.10 μM and nuclease free water. The reaction was conducted in Corbett Life Science Rotor-Gene 6000 Cycler (Qiagen, Hilden, Germany). The PCR cycling parameters were 95 ˚C for 2 min, then 40 cycles of 95 ˚C for 15 sec and 60 ˚C for 1 min.

**Histopathological examination. **Specimens from the trachea, lung and kidney were placed in 10% buffered formalin, sectioned, stained with hematoxylin and eosin (H & E) and evaluated for histological lesions.

## Results

**Clinical signs, gross lesion and mortality.** No clinical signs of respiratory infection were observed until 3 DPI (Table 1). Beginning at 5 DPI, the clinical disease was visible in all infected chicks, most severe on 14 DPI. Chicks in the infected group showed a small amount of catarrhal exudate in the trachea with slight mucosal hyperemia beginning at 3 DPI. Pale and swollen kidneys were observed on 5 to 14 DPI. No clinical signs or gross lesions were observed in the control group of chicks. No mortality occurred in either group.

**Table 1:** Respiratory clinical signs in IBV infected chicks on 1, 3, 5, 7, 14 and 21-day post infection (DPI).

Groups	1 DPI	3 DPI	5 DPI	7 DPI	14 DPI	21 DPI
1	0	0	3	3	3	3
2	0	0	2	2	2	2
3	0	0	1	2	3	2
4	0	0	2	2	2	2
5	0	0	3	3	3	2

Score definition for clinical signs: 0 = Normal breathing; 1 = slight tracheal rales after provoking the chick by moving; 2 = moderate tracheal rales without moving the chick but not continuously and 3 = marked, continuous tracheal rales.

**Fig. 1:**
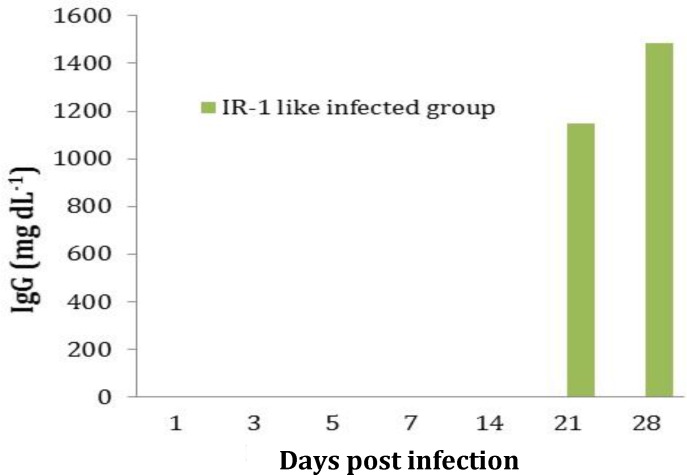
Anti-IBV antibody was measured by ELISA in serum samples of infected chicks on days 1, 3, 5, 7, 14, 21 and 28 post infections

**Anti-IBV antibody. **Antibody levels against IBV were measured in the serum from the infected or control chicks. Sera from the control group were negative for antibodies against IBV in all samples. In the infected group, no antibodies were detected on 1, 3, 5, 7 and 14 DPI, but anti-body titers on 21 and 28 DPI were 1146.50 and 1486.40, respectively. Furthermore, these values were significantly different from those of the controls (*p* < 0.05), (Fig. 1).

**Serum biochemistry. **Serum biochemical factors of the IBV infected group did not differ statistically with those of the control group (Table 2).

**Table 2:** Creatinine, uric acid, aspartate aminotransferase (AST) and alanine aminotransferase (ALT) were measured by auto analyzer in serum samples collected from control and infected chicks in different days post infection (DPI). Data are presented as mean ± SEM

**Parameters**	**Groups**	**1 DPI**	**3 DPI**	**5 DPI**	**7 DPI**	**14 DPI**	**21 DPI**	**28 DPI**
**Creatinine ** **(mg dL** ^-1^ **)**	**Control**	0.30 ± 0.10	0.35 ± 0.05	0.25 ± 0.05	0.30 ± 0.00	0.30 ± 0.00	0.35 ± 0.05	0.25 ± 0.05
	**Infected**	0.30 ± 0.03	0.38 ± 0.02	0.36 ± 0.02	0.34 ± 0.02	0.34 ± 0.02	0.38 ± 0.02	0.28 ± 0.03
**Uric acid ** **(mg dL** ^-1^ **)**	**Control**	4.25 ± 0.25	4.20 ± 0.20	4.90 ± 0.30	3.40 ± 0.20	4.20 ± 0.50	3.65 ± 0.35	4.10 ± 0.20
	**Infected**	4.30 ± 0.20	4.34 ± 0.30	4.92 ± 0.23	4.10 ± 0.07	4.60 ± 0.26	3.72 ± 0.16	4.76 ± 0.15
**AST ** **(UI L** ^-1^ **)**	**Control**	213.50 ± 6.50	200.00 ± 10.00	138.0 ± 26.00	186.50 ± 13.50	147.50 ± 2.50	173.50 ± 6.50	135.00 ± 7.00
	**Infected**	213.60 ± 8.74	204.60 ± 19.40	182.40 ± 4.48	199.00 ± 12.70	153.40 ± 15.73	175.60 ± 12.67	163.20 ± 22.30
**ALT ** **(UI L** ^-1^ **)**	**Control**	3.50 ± 0.50	3.50 ± 0.50	3.00 ± 1.00	3.00 ± 0.00	4.00 ± 1.00	4.50 ± 0.50	5.00 ± 0.00
	**Infected**	3.80 ± 0.70	3.60 ± 0.50	4.60 ± 0.40	3.80 ± 0.48	4.40 ± 0.24	4.80 ± 0.37	5.40 ± 0.24

**Real-time PCR. **The highest amount of viral RNA was detected in the trachea on 1 DPI. In the cecal tonsils and proventriculus, the titers peaked on 3DPI, while in the kidney, lung, spleen and feces, the virus reached the highest titers on 5 DPI. The viral load detected in the trachea and feces was the highest, followed by the kidney, cecal tonsils, lung, proventriculus and spleen, respectively. The virus persisted until the last day of the experiment in all tissues and feces (Table 3). 

**Fig. 2:**
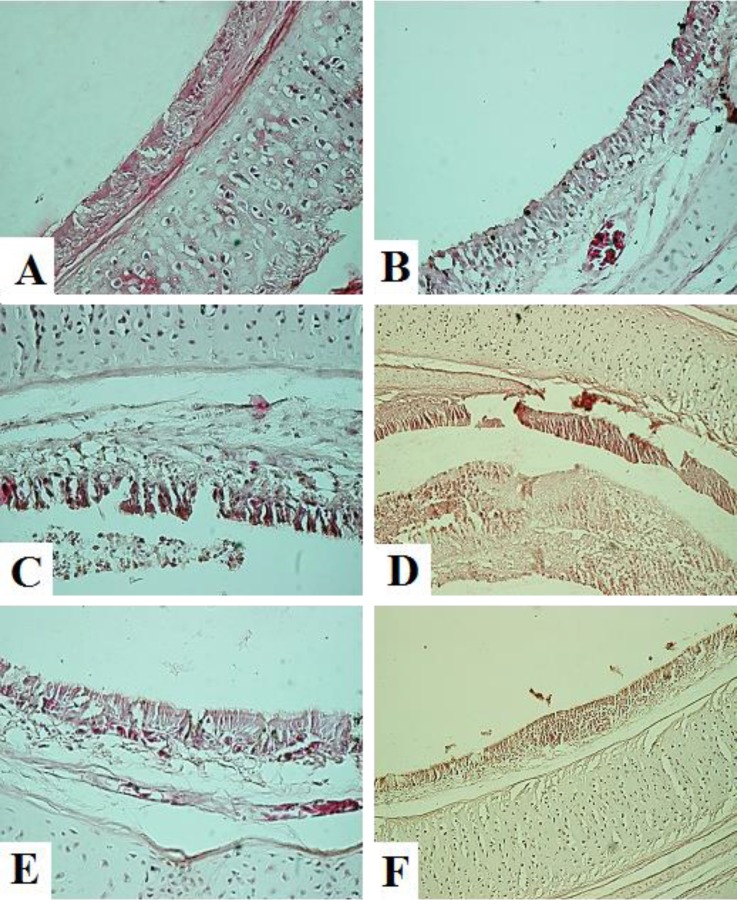
Microscopic changes in the tracheas on days 1 (A), 3 (B), 5 (C), 7 (D), 14 (E) and 21 (F) post infection in IBV infected group **A:** Normal, **B:** Mild hyperemia, **C:** Loss of cilia, mild infiltration of inflammatory cells in lamina propria, epithelial degeneration, **D:** Epithelial deciliation, degeneration, desquamation and necrosis, **E:** Epithelial hyperplasia and **F:** Severe heterophil infiltration into the tracheal mucosa (H & E; 40

**Fig. 3:**
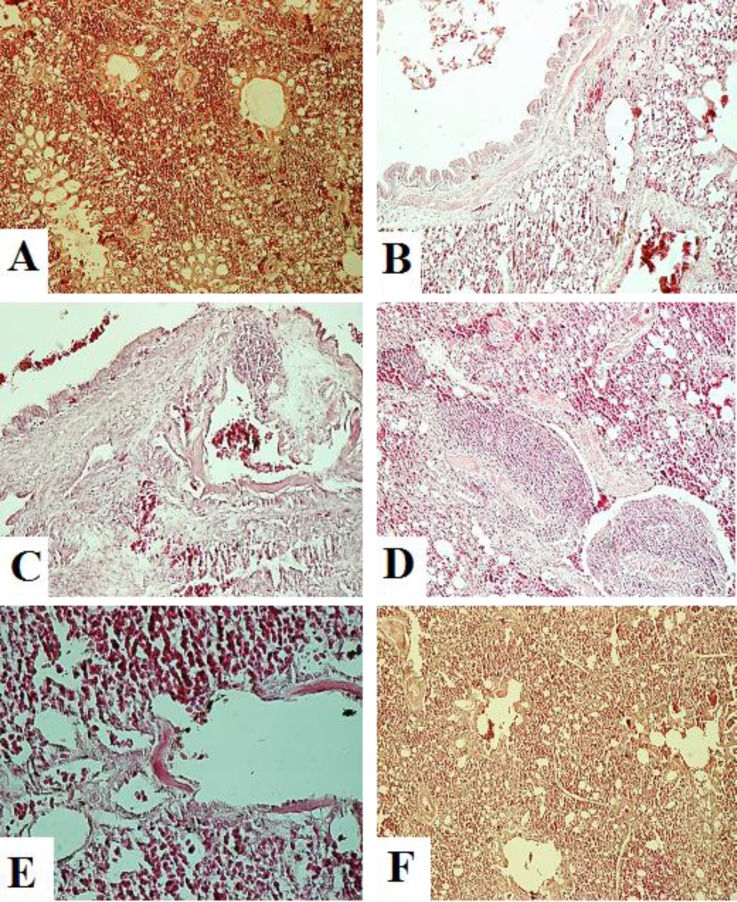
Microscopic changes in the lungs on days 1 (A), 3 (B), 5 (C), 7 (D), 14 (E) and 21 (F) post infection in IBV infected group; **A** and **B: **Bronchial Hyperemia, **C: **Degeneration and necrosis of bronchial epithelium, lymphoid nodules in bronchial wall, **D:** Lymphoid aggregation in bronchial wall, mild infiltration of mononuclear cells in interstitium, **E: **Mononuclear cell infiltration, epithelial degeneration and **F:** Hyperemia and mild inflammatory cell infiltration (H & E; 40×).

**Fig. 4:**
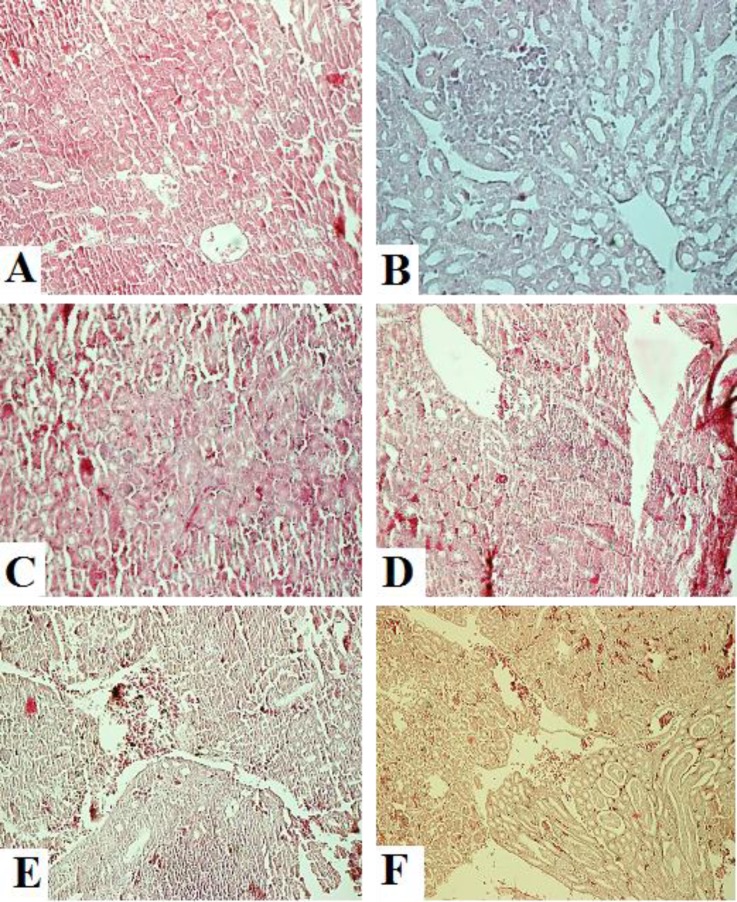
Microscopic changes in the kidneys on days 1 (A), 3 (B), 5 (C), 7 (D), 14 (E) and 21 (F) post infection in IBV infected group; **A:** No lesions, **B:** Focal mononuclear cell infiltration in interstitium, **C:** Hyperemia, swelling of tubular epithelium, **D:** Mononuclear infiltration and lymphoplasmocytic nodules in interstitium, **E:** Mononuclear cell infiltration and **F:** Mild hyperemia (H & E; 40×).

**Histopathological findings. **In the tracheas, slight congestion and edema were observed on 3 DPI. Characteristic lLesions of a degenerative stage were observed from 5 DPI. Degeneration and desquamation of ciliated epithelial and mucus-secreting cells were the most prominent findings. Epithelial hyperplasia was observed on 14 DPI and mononuclear infiltration in the lamina propria was present at 21 DPI (Fig. 2). In the lungs, hyperemia and hemorrhage were observed on 1 to 3 DPI. Desquamation of the bronchial epithelium and lymphoid nodules in the bronchial wall were observed on 5 to 14 DPI. On 21 DPI, only mild infiltration of the inflammatory cells was noted (Fig. 3). In the kidneys, focal mononuclear cell infiltration was observed in the interstitium beginning on 3 DPI. Hyperemia and edema appeared on 5 DPI. Lymphoplasmo-cyctic nodules in the interstitium appeared on 7 DPI and slight infiltration of inflammatory cells was observed on 14 DPI. The epithelium was recovered by 21 DPI (Fig. 4).

**Table 3:** Viral relative load obtained in a Q real-time PCR assay from different tissue samples and feces of IBV infected chicks, using on 1, 3, 5,7,14 and 21 days post infection (DPI). Data are presented as mean ± SEM and viral load are shown in Log10

**Days**	**Trachea**	**Lung**	**Kidney**	**Cecal tonsil**	**Proventriculus**	**Spleen**	**Feces**
**1 DPI**	7.85 ± 1.04	2.86 ± 0.45	1.93 ± 0.24	1.05 ± 0.27	2.72 ± 0.48	2.39 ± 0.42	3.38 ± 0.60
**3 DPI**	4.76 ± 0.92	4.20 ± 0.41	2.33 ± 0.23	5.41 ± 0.20	4.09 ± 0.52	2.56 ± 0.37	6.23 ± 1.16
**5 DPI**	5.72 ± 0.24	3.72 ± 0.61	3.91 ± 0.88	4.73 ± 0.26	3.45 ± 0.50	3.23 ± 0.35	7.86 ± 0.57
**7 DPI**	3.80 ± 0.29	3.26 ± 0.43	3.08 ± 0.43	3.28 ± 0.50	1.65 ± 0.19	1.92 ± 0.59	4.70 ± 0.39
**14 DPI**	1.88 ± 0.39	2.10 ± 0.33	1.48 ± 0.45	3.69 ± 0.30	0.66 ± 0.26	2.52 ± 0.29	4.71 ± 0.17
**21 DPI**	1.21 ± 0.40	0.66 ± 0.20	0.51 ± 0.17	2.45 ± 0.47	0.53 ± 0.11	1.80 ± 0.23	2.45 ± 0.52

## Discussion

Avian IBV causes a highly contagious disease in chicks. It mainly affects the respiratory tract and frequently causes damage to the kidneys and reproductive system.^1 ^New variants of IBV have emerged due to spontaneous mutation and recombination during virus replication, followed by replication of those phenotypes favored by selection.^17^

The first outbreak of IBV in Iran was in 1994 and outbreaks of 793/B were subsequently reported by several researchers.^10^ In 2012, Hashemzadeh *et al*. described a new IBV genotype in Iran,^ 11^ which was subsequently named IR-1 by Hosseini *et al.*^12^ The IR-1 has not been reported outside of Iran and there is no information about its pathogenic traits. The present study was conducted to determine the pathogenesis, tissue tropism and pathological changes of IR-1. According to our results, the virus was detected in all the tissues sampled. The viral load peaked in the lung, provenriculus and cecal tonsils by 3 DPI. In the spleen, kidney and feces, the virus yielded the highest titers on 5 DPI.

Viral load and persistence in a specific organ varied on the basis of the route of infection, duration of infection, the virus type, the chick type and other factors. Fan *et al*. compared the dynamic distribution of IBV M41, H120 and SAIBK by real-time RT-PCR in SPF chicks. The highest viral load was detected in lungs and kidneys. The M41 and H120 yielded the highest amount at 10 DPI in the lung and kidney. The relative amount of kidney infected by the SAIBK was the highest in the inoculated groups.^18^

Benyeda *et al*. compared the pathogenicity of QX, M41, and 793/B. In M41 infected group, the highest titer could be measured on 4 DPI in the trachea and cecal tonsil. The amount of 793/B was low in most of the investigated organs. Almost no RNA was found in the lung or kidney.^19^ In QX infected SPF chicks, the virus was isolated from the trachea, kidney, ovary and oviduct on 4 to 6 DPI.^20^ In the present study, the highest viral load was found in the trachea on 1 DPI, which introduced respiratory tract as the first and most suitable target organ for IBV replication. The highest viral load in the respiratory tract was observed not only in chicks infected with IBVs having respiratory affinity but also in nephropathogenic IBV infections. Hofstad and Yoder inoculated chicks with Massachusetts, Connecticut and two more unknown serotypes. Respiratory and nephropathogenic strains were found in the trachea, lung and kidney consistently for seven days following inoculation and the highest titers were found in the trachea and lungs.^21^

Histological results showed degenerative changes in the trachea and lungs, but lesions in the kidney were less severe, and there were no signs of epithelial degeneration. Since degenerative changes of renal epithelial cells are almost observed in nephropathogenic IBVs infections,^9^^,^^22^^,^^23^ IR-1 cannot be classified as a nephropathogenic strain. Our results were in agreement with Fan *et al*. who observed more severe kidney lesions in SAIBK than in M41 (non-nephropathogenic strain) infected animals. In addition, no obvious lesions were found in the kidney of the H120, an IBV strain with respiratory pathogenicity, infected group.^18^

Severe histological lesions of the trachea can occur in the nephropathogenic or QX IBV infections.^7^^,^^8^ In histological studies of Benyeda *et al*., severe lesions were found in the trachea. Lesions induced by the Greek QX and 793/B were less severe. All chicks received the QX, produced more severe kidney lesions than those infected with either M41 or 793/B.^19^ Our results were in agreement with Benyeda *et al*. who observed tracheal lesions in all infected groups but kidney lesions were less severe in M41 and 793/B infected chicks. 

In general, the clinical signs and histological analysis were in accordance with the results of real-time PCR. The peak of virus replication was recorded until 5 DPI. Thereafter, the signs of disease began to appear and histological changes were occurred gradually. Severe infection was detected during 5 to 14 DPI. Moreover, from 21 DPI, the rising anti-IBV antibody levels reduced the disease severity. 

There were some factors, which made the real-time assay less reliable in our study of pathogenicity: 1) the epitheliotropic nature of IBV, which caused broad tissue distribution and different genotypes, could replicate in the epithelial cells of respiratory tract, digestive system and renal tissue; 2) the highest load of IBVs could be detected in different organs and various time points after infection and IBVs with similar pathogenic traits might show different trends in real-time PCR and 3) high replication of virus in a distinct organ did not necessarily mean that the virus caused the most severe damage in that particular organ. Real-time PCR results demonstrated that the IR-1 tropism for the respiratory tract, digestive system and renal tissue was due to the epitheliotropic nature of IBV. 

In another pathogenicity study, Geilhausen *et al*. showed that the histopathology of the tracheas from birds exposed to the virulent and avirulent forms of IBV was markedly different while the virus isolation results were identical in both infected group.^24^

 It could be concluded that histological observations were more reliable and sensitive criteria in pathogenicity studies. Considering the severe histological changes of the trachea and lung, we estimated that IR-1 mainly affected the respiratory tract. In addition, the virus caused milder histological changes in the kidney, but these changes were not strong enough to alter the renal function. This was the first pathogenicity study of IR-1, which provided new insights on IBV pathogenesis. Middle East countries and Iran have intense trading and uncontrolled movement of inhabitants and animals across borders, which can facilitate the spread of the IR-1 to neighboring countries such as Turkey and Iraq. It is possible that this isolate exists in the neighboring countries, however, to date, it has not been identified. The complete genome sequence of IR-1 is needed to identify the origin and possible genetic recombination.
